# Whole exome sequencing may be insufficient to cover the causality spectrum of rhabdomyolysis

**DOI:** 10.1016/j.ymgmr.2018.08.001

**Published:** 2018-09-11

**Authors:** Josef Finsterer, Sinda Zarrouk-Mahjoub

**Affiliations:** a Krankenanstalt Rudolfstiftung, Vienna, Austria; b University of Tunis El Manar and Genomics Platform, Pasteur Institute of Tunis, Tunisia

**Keywords:** Mitochondrial, mtDNA, Phenotype, Genotype, Rhabdomyolysis, Whole exome sequencing, Pathogenicity

We read with interest the article by Sambuughin et al. about a genetic study of seven adult males with recurrent exercise-induced rhabdomyolysis [1]. The study raised the following comments.

We do not agree with the notion that rhabdomyolysis generally manifests with myalgia [1]. On the contrary, rhabdomyolysis is frequently a painless condition, manifesting with fatigue, exercise intolerance, and myoglobinuria.Too match, patient R465 did not complain about myalgia but about chest pain [1].

Additionally, the authors mix up electromyography with nerve-conduction studies. In patient R302 no sensory nerve action potential of the right peroneal nerve could be elicited [1], being interpreted as mononeuropathy of this nerve. Nerve-conduction studies were normal in the other six patients. Were nerves other than the peroneal nerve stimulated in patient R302? Which were the findings on needle electromyography in the seven patients?

Another shortcoming of the study is that mtDNA was obviously not covered by WES. Thus, mutations in mtDNA located genes going along with rhabdomyolysis may have been missed.

Furthermore, it is unclear which criteria were applied to assess a mutation as “pathogenic”, “likely pathogenic”, “VUS”, or “likely benign”. Were all variants detected by WES confirmed by Sanger sequencing? For assessing if a variant is pathogenic, segregation of the variant with the phenotype through generations needs to be documented [2]. Were variants or phenotypic features of the seven patients also found in their first-degree relatives? No functional data were provided to assess the effect of a variant on biological functions.

Metabolic myopathies frequently manifest as a multiorgan disease [3]. Were organs other than the muscle prospectively investigated for involvement in the underlying genetic defect?

Were any abnormalities of the acyl-carnitine profile detected in any of the seven patients in addition to patient R469?

Overall, this interesting study could be more meaningful if the above-mentioned issues would be sufficiently addressed.

## Conflict of interest

There are no conflicts of interest.

## Funding

No funding was received.

## Author contribution

JF: design, literature search, discussion, first draft, SZ-M: literature search, discussion, critical comments.
